# Linguistic and Cultural Challenges in Communication and Translation in US-Sponsored HIV Prevention Research in Emerging Economies

**DOI:** 10.1371/journal.pone.0133394

**Published:** 2015-07-30

**Authors:** Donna Hanrahan, Patrina Sexton, Katrina Hui, Jennifer Teitcher, Jeremy Sugarman, Alex John London, Mark Barnes, James Purpura, Robert Klitzman

**Affiliations:** 1 Columbia University, New York, NY, United States of America; 2 Berman Institute of Bioethics, Johns Hopkins University, Baltimore, MD, United States of America; 3 Center for Ethics and Policy, Carnegie Mellon University, Pittsburgh, PA, United States of America; 4 Harvard Law School, Cambridge, MA, and Partner, Ropes & Gray, LLC, Boston, MA, United States of America; 5 Applied Linguistics and TESOL Program, Teachers College, Columbia University, New York, NY, United States of America; 6 Masters of Bioethics Program, Columbia University, New York, NY, United States of America; Stony Brook University, UNITED STATES

## Abstract

Linguistic and cultural differences can impede comprehension among potential research participants during the informed consent process, but how researchers and IRBs respond to these challenges in practice is unclear. We conducted in-depth interviews with 15 researchers, research ethics committee (REC) chairs and members from 8 different countries with emerging economies, involved in HIV-related research sponsored by HIV Prevention Trials Network (HPTN), regarding the ethical and regulatory challenges they face in this regard. In the interviews, problems with translating study materials often arose as major concerns. Four sets of challenges were identified concerning linguistic and cultural translations of informed consent documents and other study materials, related to the: (1) context, (2) process, (3) content and (4) translation of these documents. Host country contextual issues included low literacy rates, education (e.g., documents may need to be written below 5^th^ grade reading level), and experiences with research, and different views of written documentation. Certain terms and concepts may not exist in other languages, or have additional connotations that back translations do not always reveal. Challenges arise because of not only the content of word-for-word, literal translation, but the linguistic form of the language, such as tone (e.g., appropriate forms of politeness vs. legalese, seen as harsh), syntax, manner of questions posed, and the concept of the consent); and the contexts of use affect meaning. Problems also emerged in bilateral communications – US IRBs may misunderstand local practices, or communicate insufficiently the reasons for their decisions to foreign RECs. In sum, these data highlight several challenges that have received little, if any, attention in past literature on translation of informed consent and study materials, and have crucial implications for improving practice, education, research and policy, suggesting several strategies, including needs for broader open-source multilingual lexicons, and more awareness of the complexities involved.

## Introduction

The appropriate translation of informed consent and other research-related documents, which accounts for linguistic and cultural variations, is critical to providing informed consent for research. After all, potential study participants cannot reasonably consent to research if they do not understand relevant details of the study and its implications for themselves. Nevertheless, translation of informed consent and other research documents into foreign-language contexts continues to be associated with practical and conceptual challenges.

For instance, over 80% of subjects in certain studies did not understand concepts such as placebo, randomization, and the ability to withdraw from research participation without negative consequences to themselves [1] Despite the risk for miscommunication, there is no standard method of translating clinical trial documents [2,3], though clinical researchers often use the Brislin method of forward and back translation that is performed until a satisfactory translation is achieved [4]. But many questions remain concerning the effectiveness of this approach, such as whether study participants fully understand consent forms, study purposes and procedures. While consent form readability and comprehension has been found to be suboptimal in the US [3,4,5,6] additional questions arise regarding US sponsored research conducted abroad in non-Organisation for Economic Co-operation and Development (OECD) nations [5]–whether and to what degree comprehension is reduced when many participants do not speak and/or read English well, or not at all, and how researchers, IRBs, and others might best address such potential issues.

Institutional Review Boards (IRBs) and Research Ethics Committees (RECs) play vital roles in reviewing studies, including ensuring that consent forms are adequate. Yet they have been found to face considerable challenges, such as those related to resource constraints and lack of sufficient scientific expertise [5,6,7,8,9]. The few studies that have probed these committees have, however, tended to examine aspects of the form and structures of these committees, rather than the content of their decisions. IRBs have been found to often be unsure how to proceed concerning HIV prevention trials and other areas [10].

These issues are particularly important regarding research related to HIV. The pandemic is now most prevalent in the developing world where it can be challenging for subjects to provide informed consent. For example, literacy rates are often low, making informed consent difficult to obtain [4]. Further, cultural sensitivities and taboos regarding sexuality, gender and drug use can make these topics, which may be part of the consent process, difficult to discuss.

Nevertheless, scant attention has focused on how RECs and researchers view and address challenges in translations of informed consent and other study documents.

We conducted in-depth interviews with researchers and REC members concerning ethical reviews of research protocols in the HIV Prevention Trials Network (HPTN), a global network funded by the US National Institutes of Health. In these interviews, problems with translating study materials often arose as major concerns. This paper thus presents these data, exploring the specific challenges these researchers and REC members confront concerning translation and how they have sought to address these difficulties.

## Materials and Methods

As described elsewhere [11], we e-mailed researchers, research ethics committee members and chairs associated with one or more HPTN studies. Twenty-five researchers agreed to participate, of whom 13 were based in emerging. We were able to arrange interviews with 12 of these researchers. They were engaged in a total of 7 HPTN protocols (of the 12 HPTN protocols conducted over the past two years at least in part in the developing world–representing a response among studies of 58%). Of these 12 respondents, two were also members of their REC, too. We asked to put us in contact with their IRB chair or administrator, and we thus interviewed 3 additional chairs of REC or similar committees (one of whom was also a member of the government agency REC). One REC chair declined to participate, and the others did not respond to our e-mails. We thus interviewed 15 respondents–to researchers and to REC and government agency personnel (of whom two were also researchers), using a semi-structured, in-depth telephone protocol of about one hour each, through which we sought to obtain a “thick description” [12] of these phenomena. The Columbia University Department of Psychiatry Institutional Review Board approved the study, including a process for oral consent (since the interviews were all conducted by telephone and interviewees lived overseas). Specifically, prior to the interview, we first e-mailed introductory informational letters to chairs and PIs, and at the beginning of the interview we then obtained oral informed consent from all participants (which the PI–who conducted all the interviews–documented).

As seen in Table 1, 8 interviewees were male and 7 were female; and they came from Sub-Saharan Africa (7), Asia (4), and Latin American/Caribbean (4). Interviews were audio recorded and transcribed. We coded all interviews, adapting elements from Grounded Theory [13]. Specifically, we used techniques of “constant comparison” comparing data from different contexts are compared for similarities and differences, to see if they suggest hypotheses. This technique of “constant comparison” generates new analytic categories and questions, and checks them for reasonableness. During the ongoing process of in-depth interviewing, we constantly considered how participants resembled or differed from each other and what social, cultural, and medical contexts and factors contributed to differentiation. We have included relevant qualitative data extracts in our supplemental material (S1 Data).

**Table 1:** Interviewee Demographic Characteristics.

Characteristic		N	(%)
Gender	Male	8	53%
	Female	7	47%
Role*	Researcher only	10	67%
	Chair or Member of Local or Government Agency IRB only	3	20%
	Both Researcher and IRB Chair/Member	2	13%
Region	Sub-Saharan Africa	7	47%
	Asia	4	27%
	Latin America & the Caribbean	4	27%

*Note: Some respondents had multiple roles.

Grounded theory also involves both deductive and inductive thinking, building inductively from the data to an understanding of themes and patterns within the data, and deductively, drawing on frameworks from prior research and theories. In conducting thematic content-analyses, we also triangulated methods based on the literature, described earlier. We drafted the questionnaire, drawing on prior research and published literature.

After the full set of interviews was completed, three trained research assistants (RAs) and the PI (Klitzman) conducted subsequent analyses in two phases. First, we independently examined a subset of interviews, evaluating factors that affected these respondents’ experiences, identifying categories of recurrent themes and issues, to which we subsequently gave codes. We read each interview, coding blocks of text systematically, to assign “core” codes or categories (e.g., perceived problems subjects in HPTN and other studies faced in understanding informed consent). A topic name (or code) was inserted beside each excerpt of the interview to categorize the themes being discussed. Then, we worked together to reconcile these independently developed coding schemes into a single scheme. Next, we developed a coding manual, defining each code and examining areas of disagreement until reaching consensus. We discussed new themes that did not fit into the original coding framework, and modified the manual when appropriate.

We independently performed content analyses of the data in the second phase of the analysis to identify the principal subcategories, and ranges of variation within each of the major codes. We each identified sub-themes that we then reconciled into a single set of “secondary” codes and an elaborated set of core codes. We assessed subcategories and other situational and social factors. Subcategories included, for instance, specific types of terms in consent forms that participants in HPTN and other studies had difficulty comprehending.

We then used codes and sub-codes in analyzing all of the interviews. Two coders analyzed each interview. Where necessary, we used multiple codes. We examined similarities and differences between participants, categories that emerged, ranges of variation within categories, and variables that may be involved. We probed areas of disagreement through closer analysis, and checked regularly for consistency and accuracy by comparing earlier and later coded text. Core codes and secondary codes were systematically developed and well-documented.

In this paper, we present issues concerning translation of study materials–particularly informed consent forms. Research was conducted by a primary research team, composed of the PI and three research assistants.

To protect confidentiality we have identified respondents not by country, but instead only by ID number and position as a researcher (RES), REC member (REC) and/or a member of a government agency or government REC (GOV). We have indicated when interviewees held more than one position.

## Results and Discussion

**Fig 1:**
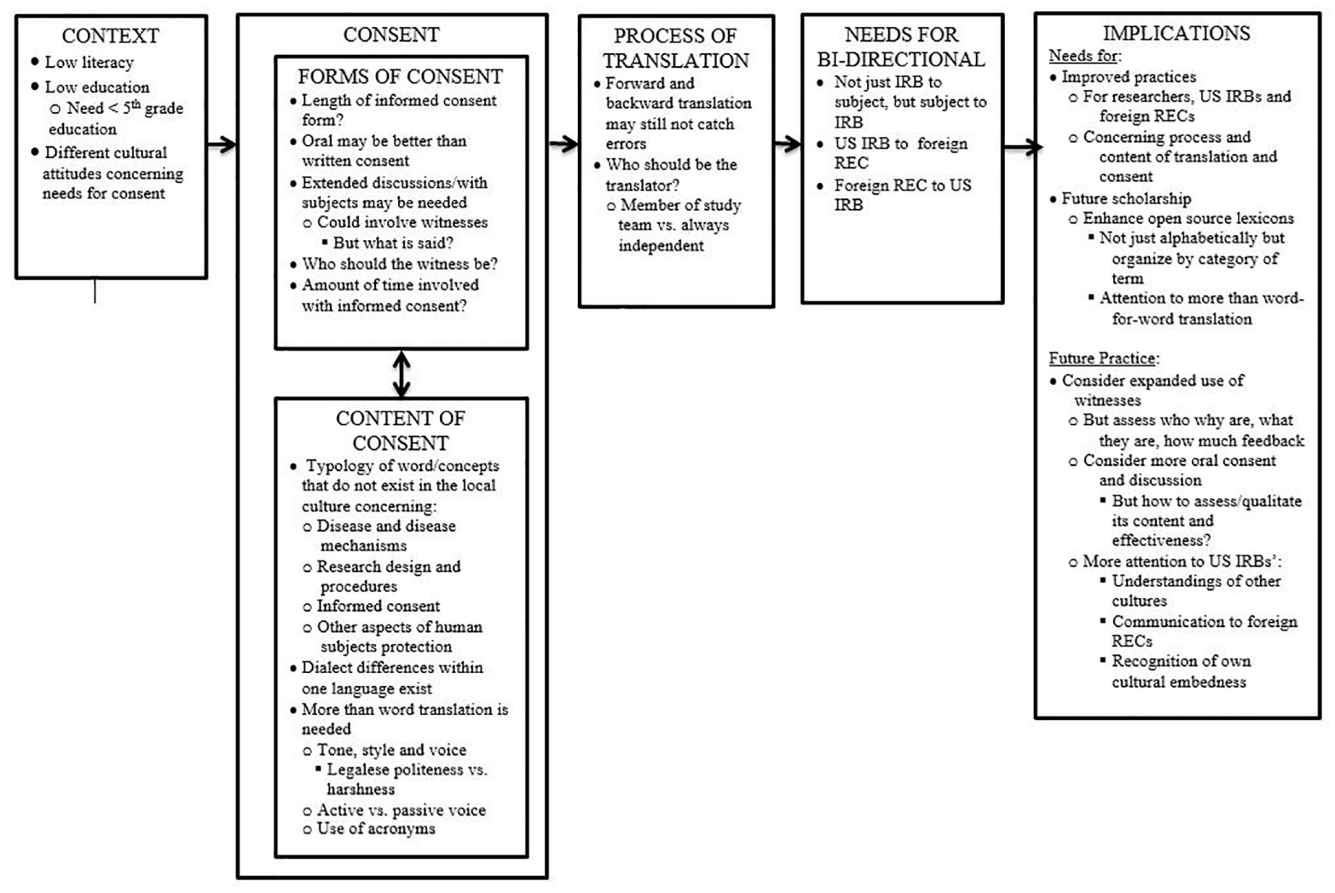
Themes concerning challenges in communication and translations regarding informed consent and other research materials in US-sponsor research in countries with emerging economies.

Overall, as seen in (Fig 1), four broad sets of challenges arose concerning translation, related to the: context; process of obtaining consent; and process of translation. All 15 respondents cited challenges stemming from language or cultural concerns particularly surrounding the informed consent process. Each interviewee discussed numerous studies that they had been involved with, and/or had reviewed.

### Context of consent

Interviewees described several challenges in obtaining consent–e.g., low literacy and familiarity with science–that were related to social and cultural contexts.

#### Low familiarity with science

Interviewees described how potential research participants may have low familiarity with research or related scientific concepts. “Placebo,” “randomization” and various other terms are “difficult for prospective subjects to understand” (RES #1), and effective communication between investigators and participants is a “big, big issue for us here.” (RES #1)

Attempts to explain study procedures can also cause confusion within a community being studied. Misunderstandings cause problems not only in obtaining consent, but also because inaccurate or misleading information about a study that can then spread in a community. One researcher described how such miscomprehension led to the “rumor that the research team was going to give the HIV virus to people, and then give them the drug.” (RES #1)

#### Low literacy

Low literacy levels can pose a series of challenges to a potential subject’s comprehension of a study in developed countries, but frequently become more significant and acute in emerging countries. In certain cultures, written informed consent is difficult “since many participants are illiterate, they can speak the language, but often not read it.” (RES #1) Furthermore, those who can read may not do so at a sufficiently advanced level to grasp major concepts and details that may be included in consent forms. “The US concept of informed consent was too foreign for what happens here. Participants can only listen to so much information at a time,” one researcher stated, “When you have a 20 page consent form, there’s a limit to how much your participant is going to grasp.” (RES #3) Both situations require that information about the protocol be delivered orally, in real time, often with linguistic modifications to accommodate the patient’s level of understanding.

US IRBs generally seek that informed consent forms are no more than an eighth grade level–though 92% of informed consent forms the US are too high [14]. As one researcher emphasized, “Informed consents written in the US are written for people who have a higher level of reading comprehension.**”** (RES #6) IRBs may thus need to adopt lower levels in certain settings. For studies in the developing country where he worked, one REC chair recommended, for reading level, “going down to fifth grade, at least,” (RES/REC #4), as the standard for study documents, to have subjects sufficiently understand them.

### Process of obtaining consent

Obstacles arise due to not only the context in which researchers seek to obtain consent but the process through which investigators try to do so. Specifically, challenges emerge concerning the possible use of oral rather than written consent, the considerable length of forms used, the extensive time that can be required to obtain informed consent, the possible use of witnesses or other third parties, and obtaining individual consent in communities.

#### Oral vs. written consent

Obtaining informed consent using the same process as that in developed countries may appear foreign or inappropriate in other cultures. Given low levels of education and literacy, written informed consent may not be appropriate. At times, written consent seemed an undue imposition of Western culture into a setting where it did not seem to make sense. As one PI explained, cultural differences could lead to accusations of colonialism:

If you follow US regulations, you need to have written consent…But there is a concept of verbal consent that is ethical and legal…But for the US, you need to have some way of documenting that verbal consent. So I was, in a way, trying to impose US regulations in a country that didn’t have them. (RES #14)

This researcher reported that in the country where he worked, oral, rather than written consent prevails in clinical settings–partly because a system exists there such that “medical providers are in this high place, and the rest of the people are very low, it’s a sort of caste-based system.” (RES #14) Based on their experiences receiving clinical care, subjects might thus feel it was inappropriate for the medical doctor to request informed consent, since consent would likely be assumed. Prevalent social hierarchies may thus foster the sense that written consent is foreign, and does not make sense.

Due to the alien nature of written consent documents in certain settings, a few researchers have used only oral informed consent. An REC chair recalled that 10 years ago, clinical practice often entailed not having any written consent, because “people don’t know how to read and write.” (RES #14) Another researcher recalled that a few years ago in a non-HPTN study,

There was no consent, it was verbal. There was even coercion: they would just test people and not tell them that they were testing them for HIV…I wanted some verification that there had been some conversation with the patient in terms of the consent for the HIV test. I was sort of adamant about that…That’s still going on in many countries, where there’s no written process of consent. (RES #14)

However, this researcher felt that there should be flexibility regarding requirements for written consent, “I’m now mellow and more flexible, and as long as somebody documents that the conversation happened, it’s OK with me.” (RES #14) As this interviewee suggested, standards and practices concerning oral consent can vary.

The practice of signing forms could also be problematic. Another researcher concurred that in such instances, to require that all participants sign the forms was “not a good way to go” (RES #1).

#### Length of consent forms

Since written consent documents are often long, some researchers and RECs appeared concerned that the subjects “may be bored before finalizing it.” (RES #15) RECs may thus ask researchers to clarify and shorten the language. As one researcher commented, “Everything could be said with fewer words. It takes too much time.” (REC # 6)

Furthermore, to translate a long form accurately into a local language is difficult. To reduce this problem, as one researcher described, a few RECs have instituted a policy of attaching “a summary, in addition to the main [document]” (RES # 5), and they encourage US IRBs to adopt shorter versions of the consent form when possible.

#### Amount of time involved in obtaining consent

Subjects vary widely in the amount of time they need to provide informed consent. “Some patients can be consented in one day,” one researcher explained, while “others must call in some relatives or other person and it can take more than one day.” (RES #6) Still, another interviewee thought it could take more than a day. As described by one researcher:

Our patients do not have the same level of understanding…If we really want participants to understand fully what is involved, we tend to spend more than one day explaining each study, to make sure the person understands fully what we intend. It's a process! (RES # 6)

#### Use of third parties and/or witnesses

Some RECs require that an independent witness observe the informed consent processes and ensue that the potential participant sufficiently understands relevant aspects of the study in order to be able to provide informed consent. One interviewee noted that if the person cannot read and write at that level, the ethics committee requires that:

Researchers have an independent witness sit in on the informed consent proceedings to make sure the participant really understands. Sometimes a translator sits in on the informed consent proceedings as well…and they’ll sit and listen to the conversation. (REC/RES #10)

Having such parties present in obtaining consent can thus be necessary and/or helpful.

Yet researchers may use these translators and/or witnesses in different ways, and to different degrees:

At the end of the discussion, I will turn to the educated witness and say, “Do you think this person is understanding what is required of him or her in it, and the data sets?” On three or four occasions, the independent witness said, “No, he doesn’t understand,” and therefore the subject is pulled out as a candidate. (REC/RES #10)

While witnesses may be asked to make such judgments about comprehension, it is not clear how he or she does so, and how valid their determinations are. Such a witness may erroneously assume that a subject sufficiently understands or not. Clearly, who the witnesses are–their own background, comprehension of English and education and training in research and health care–can affect their decisions. The interviewee above added, “The independent witness is obviously not someone who’s attached to the study site.” (REC/RES #10) But that independence may not always be the case. Questions thus surface concerning the specific extent of these individuals’ roles and skills.

#### Consent of individuals in communities

Potential subjects may view consent as more of a collective, rather than an individual activity. Researchers may thus at times need to obtain consent from not only or primarily the individual subject, but also from family or community members. Of note, cultural beliefs may differ to such an extent research design itself may need to change. For instance in one study, cultural views towards HIV testing required that researchers adjust how they approached the issue of confidentiality.

…When we talk about HIV testing, you usually test people one-on-one. I know in Africa, whenever the woman was tested first and was positive, she then had to bring back her partner–the couple assumed that whoever first tested positive brought the infection into the family. So the researchers had to modify their testing practices and test people as couples! That’s modifying our culture of confidentiality, because it was not working in that culture. (RES #14)

Researchers may thus have to modify their original research design in various ways within the context of the host culture.

#### Content of informed consent

Questions also emerged regarding the content of informed consent–specifically what exact terms to use to present complex, unfamiliar concepts regarding disease, medicine, research, and human subject protections, given that these terms and/or concepts generally do not exist in the host country language. Challenges arose concerning needs for more than literal word-for-word translation, use of colloquial and vernacular speech and dialects, overall tone and manner, and active vs. passive voice.

#### Needs for more than literal translation

Fundamental semantic concepts in one language do not always have simple one-to-one counterparts in another. Rather, the meanings of words, phrases and sentences in two languages may be similar in some respects, and vary in others. Yet research documents may not sufficiently reflect these differences. As one interviewee said, “The informed consents were an exact translation from the English language, which was difficult for us to accept at the IRB”, since it did not convey the intended meaning of the consent. (RES/REC #4) In some cases, translation adds meanings that were not present in the original, while in other cases, meanings are lost in translation due to a lack of counterpart in the original language [15]. Consequently, the lack of literal equivalence across languages presents numerous challenges for translation. Similarly, key terms may not exist in other cultures, making certain concepts hard to explain. As one interviewee noted, some languages “can’t appropriately address some of the terms” (RES # 12). As described below, five sets of difficulties arose in the data, concerning terms about 1) disease, medicine and biology, 2) research, 3) informed consent, 4) other aspects of human subject protections, and 5) tone and voice. Some of these issues can also arise within one country. An English speaker in the US with low scientific literacy may not be familiar with the concept or term “placebo control” even though he or she is a competent English speaker, but become heightened when communication occurs across different cultures.

Cultures differ in concepts of biology, disease, healing and healers. Interviewees reported having deficiencies in their languages for biological concepts such as “*virus*”, “*germ*”, “*genetics*”, “*placebo*” and “*DNA*”.

Terms about research design and procedures were also difficult to translate in fully and readily understandable ways. A researcher highlighted the need for simplification of such concepts:

We wouldn’t use something as simple as, “We’re going to take some IV samples,” or, “intravenous samples.” We would say something like, “We’re going to take some labs and some samples,” not “intravenous samples.” (RES/REC #4)

As a result, some researchers try to use communication strategies such as paraphrase to help potential participants understand complex concepts, circumlocution to facilitate understanding of complex concepts in easier terms, or metaphors to help explain complex concepts by making comparisons or associations between the complex concept and things that on the surface seem unrelated but familiar. Researchers may also add explanatory supports to define concepts. One IRB chair noted that for words that are difficult to translate or understand, like ‘randomization,’ “they always have in brackets a few words to define the concepts better.” (RES/REC #7)

Many terms used when obtaining consent relate to human subject protection can pose translation challenges. One researcher said that in his native language, “there is no word for ‘informed’ or ‘translation,’ so it has to be stated in another way.” (RES #1) Concepts may also be interpreted differently. For instance, patients from certain cultures might misinterpret the concept of “study withdrawal” in the informed consent process.

If you tell somebody, “you can withdraw from the study any time you choose to,” when you translate it locally, it comes across like: OK, we are not serious here. It conveys, to participants, a lack of seriousness about the researchers’ work. (RES #3)

That is, subjects may see this emphasis on the ability to withdraw as suggesting that the researchers do not care much if the participant remains in the study or drops out. Researchers may thus explain concepts in ways that seem straightforward to them, but unintentionally convey such other connotations.

Similarly, another researcher worked in a country in which that there was no word for “adherence.” Instead, the word used to convey this notion carried a sociolinguistic meaning of “complying,” which “implies submission.” “There are degrees of power within these words.” (RES #14) Anglicized terms may also enter into a language among educated individuals and be incorporated in consent forms and formed and back translated by local participants.

Another word that’s very difficult to translate is *empowerment*…That was one of our first obstacles. People have translated it [in a way that] doesn’t sound right or translate to *empowerment*. It sounds like an Anglicism… I would use that word, but then write in parentheses–the local term for *rescuing your control or your power*. My concept of empowerment is that *it always belongs to you*! (RES #14)

In another researcher’s country, the word *empower* also does not exist. Researchers there “use a word akin to ‘confident’”, which carries different connotations. (RES/REC #7) Given the range of these potential misunderstandings, questions arise of what standard should be used to decide when the subjects’ comprehension and consent is sufficient enough. One interviewee stated that he was worried whether subjects truly understood what they were consenting to, including the risks and benefits of participating in the study, and whether they should thus be withdrawn for an inability to give consent–i.e., partly due to translation issues.

#### Use of the colloquial, vernacular and dialects

Even within a host country, misunderstandings may also exist between RECs and subjects who may be from different educational or cultural backgrounds. For instance, IRBs and RECs may both dislike use of colloquial and vernacular terms (i.e., that spoken at home, informally, by ordinary people in a particular region, as opposed to more formal “official” language used by institutions), but not recognize the potential importance of these in communicating effectively with local subjects. Miscommunications can thus occur–e.g., regarding the terminology for sexual partners (e.g., part-time girlfriend vs. sexual partner) because the local population may use different, colloquial or vernacular terms than what many REC and IRB members may commonly use:

The REC doesn't like translations into vernacular… A lot gets lost in translation. For example, in translating the phrase “sexual partner,” one reviewer noted that the translated word choice of ‘part-time girlfriend’ was incorrect, and did not capture the idea of “full-time sexual partner”… One REC member, who’s Christian, said, the way the HPTN study described “sexual partner,” “You’re using the wrong word choice. You’re using your part-time girlfriend as opposed to your full-time sexual partner.” Our full consent tends to be at the level of someone who’s got a formal education! (RES # 10)

Yet, as the interviewee described, IRB and REC resistance to use of the vernacular can pose obstacles, since those terms may be the ones that many individuals in the local population use and best understand.

The study documents may require, too, translations of a word that has very dissimilar meanings in different dialects (i.e., focus of language “peculiar to a speaker, region or social group” [16]) of the same language. One researcher described a word that

in [one country], means *penis*. But in [in another country speaking the same language] is an *insect*. So, people will say, “Oh! There’s a [word for *penis* or *insect*]!” So people start laughing because they don’t understand. So there are a lot of regionalisms! (RES # 14)

Such barriers to informed consent can be addressed, but require close attention. As another researcher pointed out, such regionalisms can be avoided, but require recognition and care. “You have CNN in [our language]…and they have no issues with [differences in dialects], so that means we can really get over this problem.” (RES # 14) IRBs may thus try to make sure “that it’s translated into [our version of the language] and not into [another country’s version].” (RES/REC #4)

Hence, across languages, RECs and/or researchers may want consistent grammatical structure (e.g., the system and arrangement of words), which is not always possible. As a result, some interviewees felt that translations ought not to be strictly translated word-for-word in many instances, and that grammatical forms need not be always consistent. Rather, translations should prioritize the conveyance of meaning in natural-sounding language, even if this requires more flexibility in the use of different linguistic forms (“the problem is probably not just a word-to-word translation”). (RES/REC #7)

### Overall tone and manner

Challenges also arise in achieving a culturally-appropriate tone and manner. Consent forms are written in formal English, as they are conceptualized as a legal contract. Such a legalistic frame may be a mismatch and off-putting, when researchers are interested in having subjects agree to participate in a study. One researcher noted that RECs often do not like the language used in English consent forms because it comes across as culturally harsh:

The REC often wants the researchers to change the language, to make it mellow, so that it does not appear that the researchers are ‘talking down’ to the participant. For example, instead of saying “you will have to,” or, “you will give us 5 mL of blood at this visit,” they would say “we would like to request you to give…”…The REC has also always very critical of researchers saying, “we will not be liable for anything or injury” or, “you are not qualified for any compensations.” Researchers are required to say that we will give some treatment, to make the tone culturally appropriate. It becomes hard to translate, “this would be expected of you.”… Culturally, we say, “we [i.e. researchers and subjects together] will do this.” It’s almost like you are part of them; you have to make it a collective thing, rather than an individual thing. I understand that the consent has to emphasize every individual’s responsibility separately, but small changes and phrases can make things more palatable. (RES #8)

This interviewee suggested that in ordinary conversations, this kind of request would be framed with a less legalistic, more conciliatory tone; and that a legalistic tone may be inappropriate, and felt to be impersonal. Yet this respondent felt that US IRBs and researchers often fail to appreciate these differences in tone, and in the terms of polite (vs. overly harsh) discourse.

Another aspect of formal tone, and written official and legalistic documents is the widespread use of acronyms, which US consent forms also commonly include. Abbreviations of lengthy complex phrases are far less commonly used and less familiar to many participants in their usual discourse and activities. “You tend to use a lot of acronyms in English,” one chair explained, “we don’t in [our language].” (RES/REC #4) Such acronyms can potentially exacerbate participant misunderstanding, confusion, or wariness.

#### Active vs. passive voice

As described above, some grammatical structures in English do not have simple counterparts in other languages, and consequently, may not fully translate into other languages, or may require reformulation. One interviewee stated,

It’s also a grammar thing: we do have a passive voice, but we don’t use it in conversation. If we have to translate some consent from English into [our language], and we have to use passive voice, it’s not normal language that we use. (RES/REC #7)

English uses the passive voice when the agent of the action is not the focus of the sentence, as is common in scientific texts, or unknown; but interviewees felt it was generally culturally inappropriate in consent forms.

### IRB/REC misunderstandings of local practices and terms

US IRBs may insufficiently understand local cultures and may communicate poorly with not only subjects but local RECs as well. Problems thus arise concerning both subjects’ misunderstandings of Western concepts, and US IRBs’ comprehension of host cultures.

US IRBs and researchers may not understand key concepts and references in the host country culture. As a result, US IRBs may communicate poorly with not only subjects but RECs in the host country. For instance, one host country institution asked for funding for “ers.” The protocol was sent to US IRB, but reviewers did not understand what these were or why they were important. The researcher working on the study surmised that if the sponsoring organization had “on-the-ground experience” and was able to understand the reference, the IRB would have understood that a guardian shelter was “a place the facility put where guardians (i.e., unofficial support personnel, usually relatives) can go rest when they’re not by the patient bedside.” (REC/GOV #5) Here, the terms were, technically, translated correctly, but the IRB lacked local knowledge and hence was unfamiliar with the concept.

Poor communication can occur between US IRBs and most country RECs concerning not only specific words and concepts, but US IRBs’ reasons for their decisions. Underlying cultural assumptions, norms and expectations can differ, posing challenges. For instance, a cultural incongruence occurred when local researchers wanted to the use a well-known pop song in the local language in a radio advertisement to recruit subjects for a clinical trial. The researcher recalled:

The use of a song in recruitment material was deemed inappropriate by the US IRB. It is unclear why. It might have to do with the lyrics–"because we have to love each other, we are working together”. The song is [in the target language], with the music as a background. But the US IRB said, “This song is not appropriate.” (RES #1)

Presumably, the US IRB felt that the use of music and a hit song might unduly influence potential participants, and that recruitment materials should be more straightforward, fact-based and unadorned. But the US IRB did not communicate the reasons for its refusal, and the local researcher REC remained baffled and frustrated.

### The process of translation

Problems can arise during not only the process of obtaining consent from an individual participant, but also the process of translating the documents. Specifically, though researchers commonly have staff perform forward and back translation, these tasks can be highly iterative, requiring large amounts of staff, expertise and time.

#### Forward and back translation may not catch errors

US IRBs and researchers commonly use back translation to ensure accuracy, but it presents logistical and conceptual challenges. As described above, the process may not, by itself, sufficiently guarantee that researchers are conveying information in ways potential participants can adequately understand. (REC #7)

To proceed as optimally as possible, translation and back translation can be a lengthy process (“first to get it translated, and then some research networks want it back translated” [RES #8]). One researcher found no problems in translating informed consent forms from English, but that back translating everything is a, “very labor-intensive process because researchers need to have another person doing back translation–not the one who initially translated.” (RES #15)

Unfortunately, some research teams are understaffed in translators, delaying informed consent preparation. Several interviewees suggested that certain countries have a shortage of high quality translators, many of whom are already overextended. In some countries, the translators must “not belong to the project” (i.e., must be independent, but other studies may use research staff). (RES #1) Or, researchers may use the same person to do both forward and backward translations; and that individual may also in fact be a research staff member.

The process of forward and backwards translations can also require several iterations.

If I do a study for a pharmaceutical company, they send this consent translated with weird words that might be used in [one country’s dialect], but are not used in [another country’s dialect]. So it’s a back and forth for me to make them understand that people will not understand these words. (RES #14)

A researcher stated that his REC typically requires informed consent in at least three different languages that are spoken there. The researcher described the ensuing challenges related to whether the languages are being translated appropriately:

I don’t understand the languages myself, but we have 35 translators to do the translations. We send an English consent to a translator, and always give it back to the study team to look at for appropriateness, to see if it is workable for them and their participants. There are always comebacks in terms of the language not being translated appropriately. There’s nobody there to communicate with the translators to try and find the middle ground that they’re comfortable with in helping the patients understand. (RES #11)

### Local Solutions

To ameliorate these issues, some sites have also begun to develop templates for research documents, but challenges can nevertheless persist as these templates may contain legalese that their IRB or REC requires.

We already have a template here because we have been doing this for a long time. We also have some paragraphs with legal things that we cannot change, like “the university will not compensate you for damages, but will provide you care, free of charge.” We have to include these paragraphs. They are non-negotiable… But I already have a template for the consent in both languages like: the purpose of the study, how many people, the essential elements of a study, so that we can match. (RES # 14)

To try to address these problems, some researchers have also asked Community Advisory Boards (CABs) that they have established to “help modify some of the words and language” in the informed consent process. (RES #14)

## Conclusions

Though challenges concerning the translation of informed consent and other study documents in research have been discussed for over 30 years [17,18], several major problems persist. While several prior discussions have highlighted difficulties associated with translating certain words or concepts related to research studies and informed consent (e.g., placebo, randomization) [4,13,19] the on-the-ground experiences of the interviewees presented here underscore several additional sets of difficulties, related to the context, process and content of research documents, bidirectional misunderstandings and suboptimal communication, and the processes of translation itself.

Past literature about translation of informed consent and research documents has tended to focus almost exclusively on literal, word-for-word translations. Researchers have, for instance, begun to develop lexicons to aid in translation of research documents [20]. Yet our data reveal a typology of types of challenges in translation, emphasizing the importance of attending to not just the meaning of the individual words used, but also to the extended meanings, including those beyond the word level that may be implicitly conveyed, even if not explicitly expressed. These data highlight how Western concepts may not have simple counterparts in the target language and culture, and require linguistic modifications to increase comprehensibility. These data suggest how documents may thus appear to have been back translated correctly, but still cause misunderstandings or be suboptimally or inappropriately translated.

Specifically, these data suggest that misunderstandings can occur related, firstly, to terms (concerning disease and biology, research design, informed consent, and other aspects of human subject protection); and secondly, to tone and voice. This typology can aid future efforts to improve translation of study materials, by focusing endeavors, and facilitating further consideration of potential relevant terms in each category by researchers and IRBs. Cultures may traditionally have different concepts related not only to medicine and healing, but to implicit expectations concerning the roles and responsibilities of healers and patients [14,15].

Linguists and others have explored how words that translate each other are not necessarily isomorphic in meaning and function [21], “There are things that cannot be thought in another language” [22]. Human communication and interaction involves micro-level socio-linguistic features concerning when to speak and not to speak, what to say and to whom, when, where, in what manner [23].

The meaning of a particular word, for instance, may depend to a large degree on the context of other words. Language itself can in fact shape concepts that cannot be easily or well-translated from one language to another due to nuances, causing awkwardness. For example, one can say in English “take blood” or “draw blood” to express “the extraction of a sample of blood.” In Spanish, however, it would be quite odd for someone to say *necesito tomar sangre* (“I need to take blood”) since *tomar* is typically used to mean “drink” with liquids. The extraction of blood in Spanish is more accurately expressed by *extraer* (“extract”) or more familiarly by *sacar* (“take out”). Also, a Spanish-speaking physician would also probably substitute *una muestra de sangre* (“a blood sample”) for the generic term “blood” [24]. Failure of the translation process to account for these cross-cultural differences may result in miscommunication, or the mutual misunderstanding of intentions and abilities.

Consent forms may require translations of words that are not typically used in conversation, and can be awkward and/or difficult for potential subjects to understand. Also, typical of scientific language, consent forms contain numerous examples of noun compounds, consisting of two or more nouns in sequence (e.g., “substitution treatment”, “drug treatment research study”), that have no exact equivalent in many other languages, and thus often need to be rephrased (e.g., “an investigation of the use of drugs as a treatment for disease”).

The fact that consent forms are often perceived as legal contracts creates other problems, since that requires the use of certain language features to convey clarity and directness concerning the study purpose and risks, the conditions for participation, the subject’s rights, and participation. These linguistic characteristics are phonological (i.e., the sound patterns, including emphasis and stresses on particular words that can convey particular attitudes, importance or meaning), lexical, syntactic (e.g., arrangement of words in the sentence), and pragmatic. Yet these characteristics may make many participants feel mistrustful or wary.

Use of the passive voice also poses problems. Consent forms may contain sentences such as, “This is a drug that has been approved for treating drug users in the US” that use the passive voice because the person doing the approving was unknown and unimportant in this situation. Consent forms often contain sentences such as, “The treatment you are given will be decided by chance”–in which the focus is more on the treatment and the decision process than on the people who are giving the treatment or making the decisions. Hence, the passive in English is used.

English does not share these uses of the passive voice with many languages. Other languages may use to express similar meanings different structures, such as reflexives or impersonal expressions (e.g., “one will give you a treatment”), creating translation challenges. These issues can occur within a single linguistic community as well, but get compounded when translating documents across different cultures, due to differences in norms, education and other factors.

These data highlight challenges concerning not only the content, but the methods and the formal aspects of the consent process. In many instances, oral consent may be appropriate. However, oral translation, and discussion about consent, including responses to participants’ questions that may arise during the consent process, can consist of casual conversations that employ metaphors, idioms and vernacular. Yet extensions of meanings can result from the unintentional use of idioms or metaphors (e.g., “Don’t worry about the blood test; it’s a piece of cake”), and have connotations that are only derivable from the context in which the word is used (e.g., “the results are positive”–meaning “not good”). These implied connotations can relate to contextual meanings (e.g., routine expressions that only make sense in context–e.g., “what’s up?”), sociolinguistic meanings (e.g., the appropriate ways to be formal or informal), sociocultural meanings (e.g., appropriate ways to be polite in a particular culture), and psychological meanings (e.g., appropriate ways to encode attitude, emotionality, tone, stance) [25,26]. A translator’s lack of nuanced understanding of a certain dialect and different educational level from that of participants can lead to inconsistencies in translation. Kaufert and Koolage described in 1984 how native Canadian medical interpreters in clinical encounters may themselves serve as “culture brokers” and experience role conflicts [27]. The current data highlight how such cultural brokering may occur concerning research today in several regions of the world.

These data also suggest challenges concerning what oral consent should include–whether to standardize or assess the informal conversations that may ensue between participants and researchers, and if so, how. These findings suggest needs for empirical studies of what researchers say to subjects when oral communication is used, either alone to obtain consent or in conjunction with a written document.

This study highlights, too, the potential benefits and importance of having independent witnesses involved in the consent process. Witnesses have received a relatively small amount of attention in the past scholarly literature. An independent witness can potentially improve informed consent [28], yet several questions have been raised concerning the scope of their responsibilities–who they should be, and how they should be selected (e.g., whether they could be family or research team members), and what potential benefits and risks they might present in recruiting particular groups of participants in particular studies, to avoid either constituting a meaningless ritual, or intimidating potential participants [29].

Western researchers and IRBs may also not recognize in advance how frequently extended periods, even whole days, may be needed to obtain consent–when, with what proportion of subjects, and how, and exactly what information to present.

These data suggest, too, problems of bidirectional translation. US IRBs may not only misunderstand key aspects of the local language and culture, but fail to appreciate this misunderstanding and its extent, and how much potential participants may misunderstand study documents. US IRBs now seek to have consent forms be at no more than an eighth grade reading level, but fail to do so 92% of the time [12]. Yet these data suggest that in many developing countries, a far lower level (e.g., fifth grade) may be needed, though conveying a study’s complexities at that level can pose considerable challenges.

Western researchers and IRBs need to be acutely aware, too, of contrasting views regarding even the perceived value of consent, and should consider carefully how to present and frame its importance–perhaps doing so orally in ways that these IRBs and investigators script and review.

Our data have several implications for future efforts to enhance participant comprehension. Mack et al. used 12 focus groups with women in Tanzania to grasp their understandings and preferences concerning research terms in HIV studies [30]. The present data, highlighting the challenges in translations of materials for research studies, suggests that such approaches can be very valuable. Ramirez et al., in conjunction with the Population Council [31], published a lexicon that consisted initially of 49 specific terms related to sexual behavior, reproductive health, infectious diseases and research, and translating these terms into Swahili [20]. This lexicon has been expanded, and some of these words have been translated into Xhosa, Setwana, Zulu and Thai (though not all terms have been translated into all of the languages) [20].

Our findings suggest how such efforts should be broadened to include additional words, as well as discussions of common linguistic structures in consent forms (e.g., passives, noun compounds, tenses) that may not have equivalents in other languages.

The lexicon by Ramirez et al. presents words alphabetically, but might also be divided by categories of words or concepts–e.g., following the typology presented above involving categories of disease, disease mechanism, research design and subject population (including, for example, terms such as intravenous, control group, data, DNA and genetics) to help to ensure thoroughness, and facilitate the further development of such efforts. Lexicons could also be expanded to include additional languages of the 6,000 spoken today [32] and dialects.

Another possible model would be to develop an online dictionary similar to WordReference [33]–an open source dictionary and forum focused on words and phrases in 15 languages that are difficult to translate. WordReference does not, however, currently include many terms used in research, or many languages spoken in countries with high HIV prevalence.

Reference tools could also be developed to contain template language that is both amply back translated and culturally appropriate for a wide variety of host countries.

Our data also suggest that such an expanded lexicon could help with some, but clearly not all of the problems identified in these data. A word-for-word lexicon may not be readily capture contexts that can be crucial in interpreting the meanings of particular words. The lack of direct literal translations can also substantially increase the length of informed consent forms (e.g. the two-word phrase, “to abstain,” is translated in the lexicon by Ramirez et al. with 20 word phrases [20]).

Not only specific terms, but other key components of communication related, for instance, to tone, voice and the use of acronyms, should be included. For example, while in English the notion of tense denotes when an action is taking place, and temporality is fundamental to making sense of events, other languages (e.g., Javanese, Burmese or Malay) do not have grammatical tense. Thus, guides should be developed to accompany lexicons to include relevant in-depth information about more than word-for-word translations–concerning appropriate tone.

These data pose larger questions, too, concerning the tone that consent forms should use. Many IRBs seek to employ language and tone that they feel is appropriate and professional, but that subjects may feel is too legalistic, harsh, or impolite. Interviewees suggest that US IRBs often seek to avoid use of colloquial or vernacular language. But at times, colloquial language may aid subject comprehension; and the boundary between vernacular discourse may be blurry, rather than rigid. In many instances, both types of discourse may be beneficial and used by subjects. Some local researchers thus put various terms in brackets. Yet, US IRBs may sometimes fail to appreciate these complexities, and may not want to include more than one possible translation of a word. Further research should explore how often and when researchers add such terms, how doing so may aid subject comprehension, and how IRBs respond.

These data also underscore how the process of translation can be very time and labor intensive. Researchers should thus plan adequate time, and be fully aware that back translations may not always capture problems. On-line hyperlinked informational platforms in which subjects should click for more information about any particular topics might also be helpful and worth developing. These efforts may also require additional resources. USAID [34] funded the Ramirez et al. lexicon through 2015. Other organizations should consider helping to fund such efforts, especially as new interventions and approaches toward intervention, prevention and treatment of HIV–as well as other disorders (e.g., TB, malaria)–are established over time.

These data also elucidate challenges in bidirectional cultural communication and translation from local languages to Western cultures. At times, US IRBs may need to communicate more effectively explanations for their decisions to foreign RECs (e.g., when a local song many not be appropriate to use). US IRB make decisions for reasons that may be culturally specific in ways that foreign IRB may not understand. US IRBs could thus seek to improve communication with not only foreign participants, but foreign RECs through appropriate cultural translation is needed of IRB memos, too.

These data highlight as well needs for broader open discussions concerning how informed consent forms can both satisfy the needs of a legal system in Western cultures and account for cross-cultural sensibilities. These interviews underscore critical questions concerning whether at times Western notions of informed consent may represent ideals that in current practice may not always be fully attainable. Clearly, researchers should still pursue these ideals as much as possible, but may not always fully succeed [35].

These data further highlight the need for heightened connections between research ethics and linguistics, which appear to have been limited heretofore. Much of the literature concerning theories of translation has focused on works of literature, and the difficulties of achieving “faithful” translation [12]. Prior research in linguistics focused on other realms of communication, but not focused on the field of research ethics. The data here suggest needs for much more work in this area.

US IRBs may also need to recognize more fully their own cultural embeddednes–how much they themselves may make certain assumptions that foreign study participants and RECs may not share.

This study has several potential limitations. These data are based on a relatively small number of in-depth interviews with investigators who conduct HIV studies in developing countries and REC personnel outside the US; however, future studies can examine these issues among larger samples of investigators, REC personnel and others in these and further countries, examining research on other disorders, too. However, the experiences here suggest that obstacles exist–e.g., in contacting and entering into studies REC members who have reviewed specific protocols. Of the 15 respondents, 5 were REC members or chairs. Though many of these interviewees are researchers, their first-hand experiences and perspectives are vital to understand. The number of themes raised in these data may seem relatively high, but these respondents each ended up discussing a wide range of topics and protocols. These data also explore these interviewees’ experiences and perspectives at one point in time, but future studies should investigate attitudes prospectively over time and do so among larger numbers of participants to assess whether their views alter over time.

In sum, the data from the current study suggest a variety of challenges that US sponsors and researchers doing research abroad face. As clinical trials in emerging economies, related to prevention and treatment of HIV and other disorders become more common, fuller, deeper and more nuanced understandings of the bilateral, linguistic and cultural challenges involved are crucial. These understandings can help in the development of improved researcher and REC practices in translating and obtaining informed consent, and enhancing other aspects of studies.

## Supporting Information

S1 Data Relevant Qualitative Data Extracts. (DOCX)
