# Supplementary material for: Linguistic and Cultural Challenges in Communication and Translation in US-Sponsored HIV Prevention Research in Emerging Economies
Source: PLoS One. 2015 Jul 30;10(7):e0133394. doi: 10.1371/journal.pone.0133394 (PMC4520713; doi:10.1371/journal.pone.0133394)
Supplement: S1 Data — (DOCX) [file pone.0133394.s001.docx]

**S1: Data: Relevant Qualitative Data Extracts**

***CONTEXT ISSUES***

- **Low literacy**
  - The same when we translate the correct word, but if it’s going to be an informed consent, then it’s at a level for everyone to understand, probably I would say more informal or like the way they used to use the word. It’s what is needed. So, basically I would say the level -- not the words, but the level. Going down to fifth grade, at least. **RES/REC # 4**
  - The need to have participants sign consent forms is difficult since many participants are illiterate. They often can speak the language, but not read it. **RES # 1**
  - The US concept of informed consent was too foreign for what happens here. They can only listen to so much information at a time. When you have a 20 page consent form, there’s a limit to how much your participant is going to grasp, even when you use a checklist to assess how much information they grasp. **RES # 3**
- **Low education**
  - “So we have many big issues in terms of education inside the village. It may be not a good way to go, to sign the forms so we need to explain to them that how we are going to overcome that difficulty for some participants. **RES # 1**
  - “Informed consents written in the U.S. are written for people who have a higher level of reading comprehension. Consent used in the United States is OK for persons with a high education level. **RES # 6**
- **Low exposure (to research or concepts)**
  - It would be hard for them to understand ‘Placebo’ or ‘randomization’, that’s a big, big issue for us here.” There is no word for ‘informed’ or ‘translation’, so it has to be stated in another way; there’s been a lot of flexibility. **RES # 1**
- **Process issues about informed consent (&maybe about research?)**
  - **Verbal vs. Written**
    - “I had a study many years ago, a mother-to infant transmission study where we were going to treat women. So for the HIV testing -- in the general practice of medicine in the country, they don’t take any written consent for anything there, nothing was written. Because, either people don’t -- there’s not enough people who read and write, or the system is such that medical providers are in this high place, and the rest of the people are very low. It’s sort of a caste-based system. We talked about the specific consent for HIV testing. I could not understand that. And they insisted I went there to train people, to set a protocol, and I realized that there was no consent, it was verbal. There was even coercion: they would just test people and not tell them that they were testing them for HIV…I wanted some verification that there had been some conversation with the patient in terms of the consent for the HIV test. I was sort of adamant about that... That’s still going on in many countries, where there’s no written process of consent.” **RES # 14**
  - **Length**
    - We started coming up with the summary, in addition to the lengthy vision of the proposal. So now, there usually is a summary, in addition to the main proposal. **RES # 5**
  - **Community group vs. individual**
    - **Witness:** “Researchers have an independent witness sit in on informed consent proceedings to make sure the participant really understands. Sometimes a translator sits in on the informed consent proceedings as well…And they’ll sit and listen to the conversation. And there might even have to be a third person in who then can provide translation. Because unfortunately, my only language is English. At the end of the discussion, I will turn to the educated witness and say, “Do you think this person is understanding what is required of him or her in it, and the data sets?” On three or four occasions, the independent witness said, “No, he doesn’t understand,” and therefore [the subject is] pulled out as a candidate. The independent witness is obviously not someone who’s attached to the study site.” **REC/RES # 10**
  - **Community advisory boards (CABs)**
    - We have a community advisory committee. I use my CAB to help modify some of the words and the language that they put in. But, if I do a study for a pharmaceutical company, they send this consent translated with weird words that might be used in [one country’s dialect] but are not used in [another country’s dialect]. So it’s a back and forth for me to make them understand that people will not understand these words. **RES # 14**
    - I think people should know what they are being tested for. Whether it’s written or not, I’m now mellow and more flexible, and as long as somebody documents that the conversation happened, it’s OK with me. I can give you another example with HIV testing, and that’s the confidentiality issue. When we talk about HIV testing, you usually test people one-on-one. I know in Africa, whenever the woman was tested first, if she was positive, she then had to bring back her partner – the couple assumed that whoever first tested positive brought the infection into the family. So the researchers had to modify their testing practices and test people as couples! That’s modifying our culture of confidentiality, because it was not working in that culture. **RES # 14**

***CONTENT ISSUES***

- **Words/concepts that do not exist in the local culture**
  - Sometimes they force us to use other words; like I have an adherence study and there are four languages where they wanted us to define “adherence,” and the word “adherence” is very difficult to translate into Spanish. There’s no right word to use in Spanish for “adherence.” So that was one of our first obstacles -- how are we going to define “adherence” so that these participants and the IRB understand? I would use an Anglicism which is not a real because the other words that you could use have other implications. So, that’s why translating the word adherence is difficult. **RES # 14**
  - Sometimes there is no specific word in [our language] for words in English. Furthermore, grammatical structure does not fully translate well. For example, the word “empower” does not exist in [our language]. The translated version is not a word typically used in conversation and is not easy to read. Translations use a word akin to “confident.” **RES/REC # 7**
- **Understanding of words about disease and disease mechanisms; E.g., virus, germ**
  - We wouldn’t use something as simple as “We’re going to take some IV samples,” intravenous samples.” We would say something like, “We’re going to take some labs and some samples,” not “intravenous sample.” Simple things like that. The thing is that our IRBs are a mix of members. We have a mix of members. Right now, I think I’m the only M.D. in my panel, in particular. Basically, we have people from the social sciences, public health, other professions, and those issues usually come up. For our institution, it’s very simple to say, “Yes, we’re going to take some intravenous samples,” but then they react immediately, “no, that’s not really the way”. **RES/REC # 4**
  - “It’s just that the concept of a virus, or the concept of a germ may not always be plain in the language, there may not be a word for it in the language.” **RES # 12**
  - Furthermore, concepts like “placebo” and “randomization” are difficult to explain to subjects. **RES # 1**
- **Understanding about scientific design/science E.g., IVs, placebo, randomization**
  - “Placebo” and “randomization”…they always have in brackets a few words to define the concepts better. Also, scientific words like DNA do not translate well. **RES/REC # 7**
- **Understanding about process of the informed consent or research process**
  - “One of the things that is most confusing is that when we start talking about risk in terms of HIV, you introduce the fact that some might be participants in other studies related to HIV, every time something of that sort comes into the informed consent, that’s very confusing.” **RES/REC # 4**
  - If you tell somebody “you can withdraw from the study any time you choose to”, when you translate it locally it comes across like: “OK, we are not, we are not serious here”… It conveys to participants, a lack of seriousness about the researchers work. **RES # 3**
  - The informed consents were an exact translation from the English language, which was difficult for us to accept at the IRB because no cultural pieces were involved. We called the researcher’s attention to that and hired a new coordinator to work that out. **RES/REC # 4**
- **Cultural Informed Consent Concerns**
  - I cannot justify accepting or allowing behavior because if it’s mistreatment or abuse. But there are subtleties of that, of course. Certainly, the U.S. cannot impose certain practices. For example, the specific example of written informed consent. **RES # 14**
- **Overall Manner (Tone/Voice)**
  - **Active/Passive Voice**
    - I think there are many words. The problem is probably not just word to word translation; it’s also a grammar thing: we do have passive voice, but we don’t use it in conversation. If we have to translate some consent from English into [our language], and we have to use passive voice, it’s not normal language that we use. Most researchers want to keep it in passive voice because the protocol says that it has to be best translated with the original English. **RES/REC # 7**
  - **Acronyms**
    - “Related to HIV… you tend to use a lot of acronyms in English. We don’t, in [our language]’ **RES/REC # 4**
- **Other Problems**
  - **Forward and back translation may still not catch errors**
    - In many countries an informed consent document is very confusing. Many words do not translate well into the language. There are words that don’t translate well into my language, that are involved in research. Problem is that it’s very general words**. RES # 13**
    - It becomes a very labor-intensive process because researchers need to have another person doing back translation, not the one who initially translated it. **RES # 15**
    - Templates: “We already have a template here because we have been doing this for a long time. We also have some paragraphs with legal things that we cannot change like, “the university, will not compensate you for damages, but will provide you care, free of charge”. We have to include these paragraphs. They are non-negotiable… But, I already have a template for the consent in both languages like: the purpose of the study, how many people, the essential elements of a study, so that we can match. , because that’s another issue that you don’t have. For my IRB, I have to submit the English and the translated consent form, and sometimes the IRB compares them. That can be bad, because if you miss something, and there’s one sentence in one and not in the other, that’s a red flag for them. **RES # 14**
  - **Different Dialects/ Intercultural differences within one language**
    - We make sure the consent form is a really informed consent. That it’s clearly understood, that it’s translated into [one country’s dialect] and not into [another country’s dialect]. **RES/REC # 4**
    - “International Television networks that are in our language are seen all over the world. But their anchors and their people know which words to use. There is a way to speak [our language] in a way that everybody understands, but you have CNN in [our language] …and they have no issues with the [differences in dialect], so that means that we can really get over this problem.” **RES # 14**
